# Using a Virtual Reality CAVE–Based Mindfulness Intervention to Promote Mental Well-Being in Adolescents With Anxiety Symptoms: Pre-Post Mixed Methods Pilot Study

**DOI:** 10.2196/91819

**Published:** 2026-06-12

**Authors:** Clare Tsz Kiu Yu, Alex Pak Lik Tsang, Kelly Ka Yu Chan, Irene Oi Chiu Fung, Zoe Suk Yi Ngai, Lester Yin Cho Kwok, Sam Kam San Chan, Gary Tsun Fung Fung, Sammy Law Sun Li, Victor Wai Keung Ching, Patrick Pui Kin Kor

**Affiliations:** 1 Division of Psychiatry, University College London, London, England, United Kingdom; 2 School of Nursing, The Hong Kong Polytechnic University, GH506, Hung Hom, Kowloon, China (Hong Kong), +(852) 27664369; 3 Department of Early Childhood Education, The Education University of Hong Kong, Hong Kong, China (Hong Kong); 4 The Friends of Scouting, Scout Association of Hong Kong, Hong Kong, China (Hong Kong)

**Keywords:** mindfulness, adolescent, virtual reality, mental health, anxiety

## Abstract

**Background:**

Adolescent anxiety is a growing public health concern associated with significant social and emotional impairment. Mindfulness-based interventions (MBIs) have shown promise in reducing anxiety and improving well-being; however, engagement remains challenging. Virtual reality (VR)–based delivery may enhance immersion and attention, potentially addressing barriers of traditional mindfulness formats. Evidence on VR-based mindfulness interventions for adolescents, particularly in Hong Kong, remains limited.

**Objective:**

This study aimed to evaluate the feasibility and acceptability of a VR-MBI delivered via a CAVE, an enclosed VR environment with three projected walls displaying immersive natural scenes and ambient sounds, for adolescents with mild-to-moderate anxiety symptoms in Hong Kong. Secondary aims were to explore preliminary effects on psychological outcomes and physiological stress regulation and to identify facilitators and barriers to engagement.

**Methods:**

A mixed methods, single-group pre-post study was conducted with adolescents experiencing mild-to-moderate anxiety symptoms, recruited from secondary schools and youth service organizations in Hong Kong. Participants completed an 8-week group-based VR-MBI. Feasibility and acceptability were assessed using recruitment, attendance, retention, homework practice frequency, dropouts, and adverse events. Psychological outcomes were measured using the Depression Anxiety Stress Scale–21 and the Mindful Attention Awareness Scale. Heart rate variability indices, including the standard deviation of normal-to-normal intervals and root-mean-square of successive differences, were collected at baseline and postintervention using a wearable device. Focus group interviews explored participants’ experiences. Paired-sample *t* tests and Wilcoxon signed rank tests examined pre-post changes, and qualitative data were analyzed using thematic analysis, with findings integrated through triangulation.

**Results:**

A total of 42 participants (mean age 14.88, SD 1.90 years; 20/42, 47.6% female; 22/42, 52.4% male) enrolled and completed both assessments. Attendance was high, with 73.8% (31/42) of participants attending at least 80% (8/10) sessions, and participants engaged in regular homework practice. No dropouts or adverse events were reported. No significant pre-post changes were observed in self-reported distress, anxiety, depression, stress, or trait mindfulness (all *P*>.05). However, significant improvements were observed in both heart rate variability indices, standard deviation of normal-to-normal intervals (mean difference 17.6 ms, 95% CI −33.88 to −1.32; *P*=.04; Cohen *d*=0.38) and root-mean-square of successive differences (mean difference 20.20 ms, 95% CI −38.76 to −1.65; *P*=.03; Cohen *d*=0.39), which may suggest preliminary enhancements in physiological stress regulation. Qualitative findings suggested perceived benefits in emotional regulation, stress reduction, focus, and sleep, with the immersive environment and group-based format identified as key facilitators.

**Conclusions:**

The CAVE-based VR-MBI was feasible and acceptable for adolescents with mild-to-moderate anxiety symptoms in Hong Kong. Despite no significant changes in self-reported outcomes, physiological improvements and positive qualitative feedback suggest early benefits not captured by self-report measures. These findings support further investigation of using controlled designs and longer follow-up periods.

## Introduction

Anxiety is a growing concern among Hong Kong adolescents, with recent school-based data indicating that 11.9% meet criteria for an anxiety disorder [1]. When left untreated, youth anxiety is associated with poor academic functioning, social functioning, and well-being [2-5], and increased risk of depression, substance misuse, suicide attempts, and hospitalization in adulthood [6-8]. Mindfulness-based interventions (MBIs), structured programs cultivating present-moment awareness with openness and nonjudgment [9,10], have emerged as a promising preventive approach, with evidence indicating moderate reductions in anxiety, and gains in emotional regulation, resilience, and coping among young people [11]. Local evidence further supports the feasibility, acceptability, and effectiveness of MBI for Hong Kong’s adolescents, with studies reporting improvements in emotion regulation, working memory, and reductions in stress and depressive symptoms across diverse school populations [12-14]

Despite these promising outcomes, traditional MBIs pose challenges for adolescents, who often experience mind-wandering, intrusive thoughts, classroom distractions, and boredom during mindfulness practice [15,16], highlighting the need for more engaging and immersive delivery formats. Virtual reality (VR) has emerged as a promising alternative, offering an immersive environment theorized to enhance engagement and sustained attention [17-19]. Evidence suggests VR-delivered mindfulness interventions outperform traditional formats in adherence [20] and show comparable or superior improvements in anxiety, mindfulness, and positive affect [21], with a recent review reporting small-to-large improvements across mental health outcomes [22]. Among adolescents, VR-based well-being interventions have been shown to be feasible and acceptable [23], and a 3-week VR-MBI demonstrated reductions in stress and depressive symptoms among adolescents in the United States, although headset-related discomfort, such as nausea, has been reported [24].

To date, research on VR-MBI for adolescents remains limited, and no published studies have focused on adolescents in Hong Kong. This study addresses this by evaluating the feasibility and acceptability of a VR-MBI for adolescents with mild to moderate anxiety symptoms in Hong Kong. Given prior reports of headset-related discomfort, we designed our VR program using CAVE technology—an immersive VR system that projects virtual environments onto three surrounding screens without requiring participants to wear head-mounted displays.

This study aimed to (1) examine the feasibility and acceptability of VR-MBI among adolescents with mild-to-moderate anxiety in Hong Kong; (2) explore preliminary effects on mental well-being outcomes, including stress, anxiety, depression, and distress; and (3) identify key facilitators and barriers influencing adolescents’ engagement with and implementation of the VR-MBI.

## Method

### Research Design

The study used a mixed methods, single-group pre-post design involving self-reported questionnaires, focus group interviews, and stress-related biological markers collected via a digital watch before and after the intervention. The recruitment began in October 2024, with the intervention and data collection conducted from October 2024 to August 2025. This study was reported in accordance with the Transparent Reporting of Evaluations with Nonrandomized Designs checklist.

### Ethical Considerations

Ethical approval for this study was granted by the Institutional Review Board of The Hong Kong Polytechnic University, Hong Kong (HSEARS20240326009), in accordance with relevant ethical guidelines for human research, including the Declaration of Helsinki.

Participation in the study was entirely voluntary. Prior to enrollment, researchers explained all study procedures, provided a participant information sheet, and clearly outlined participants’ rights, including the right to withdraw at any time without consequence. Written informed consent was obtained from all participants prior to enrollment. For those under 18 years of age, consent was also obtained from their legal guardian.

To protect participants’ privacy and confidentiality, all identifiable information was anonymized prior to data analysis. The code list linking participants’ real names to their assigned codes, along with signed consent forms, was stored separately from the anonymized data. All electronic data were stored securely on the institution’s password-protected, encrypted drive, accessible only to core research team members.

Participants received a theme park ticket (approximately US $50) as compensation for completing the pre- and postintervention assessment and interviews.

### Participants and Sample Size

Participants were adolescents in Hong Kong presenting with mild-to-moderate anxiety symptoms. Inclusion criteria were:

1. Currently enrolled in secondary education in Hong Kong.
2. Experiencing mild-to-moderate anxiety symptoms, as determined using the Generalized Anxiety Disorder Scale (GAD-7; operationally defined as a score of >10) or identified through referrals from social workers or teachers.

Participants were excluded if they were currently taking psychotropic medication or had a diagnosed mental health condition requiring specialized or intensive clinical care, because the program was designed as a low-intensity, nonclinical intervention and was not intended to replace individualized clinical treatment.

A sample size of 40 participants was predetermined based on recommendations for pilot studies to estimate feasibility outcomes and preliminary intervention effects [25].

### Intervention

We designed VR-MBI as a program that aimed to help participants recognize the links between bodily sensations, feelings, and thoughts related to anxiety. It integrated mindfulness-based cognitive therapy principles, present-moment awareness, and nonjudgmental acceptance to interrupt habitual emotional and cognitive patterns [26,27], and mindful parenting principles adapted for adolescents—self-compassion, curiosity, and intentional responding—to promote calmer, more reflective reactions [28].

**Figure 1.**
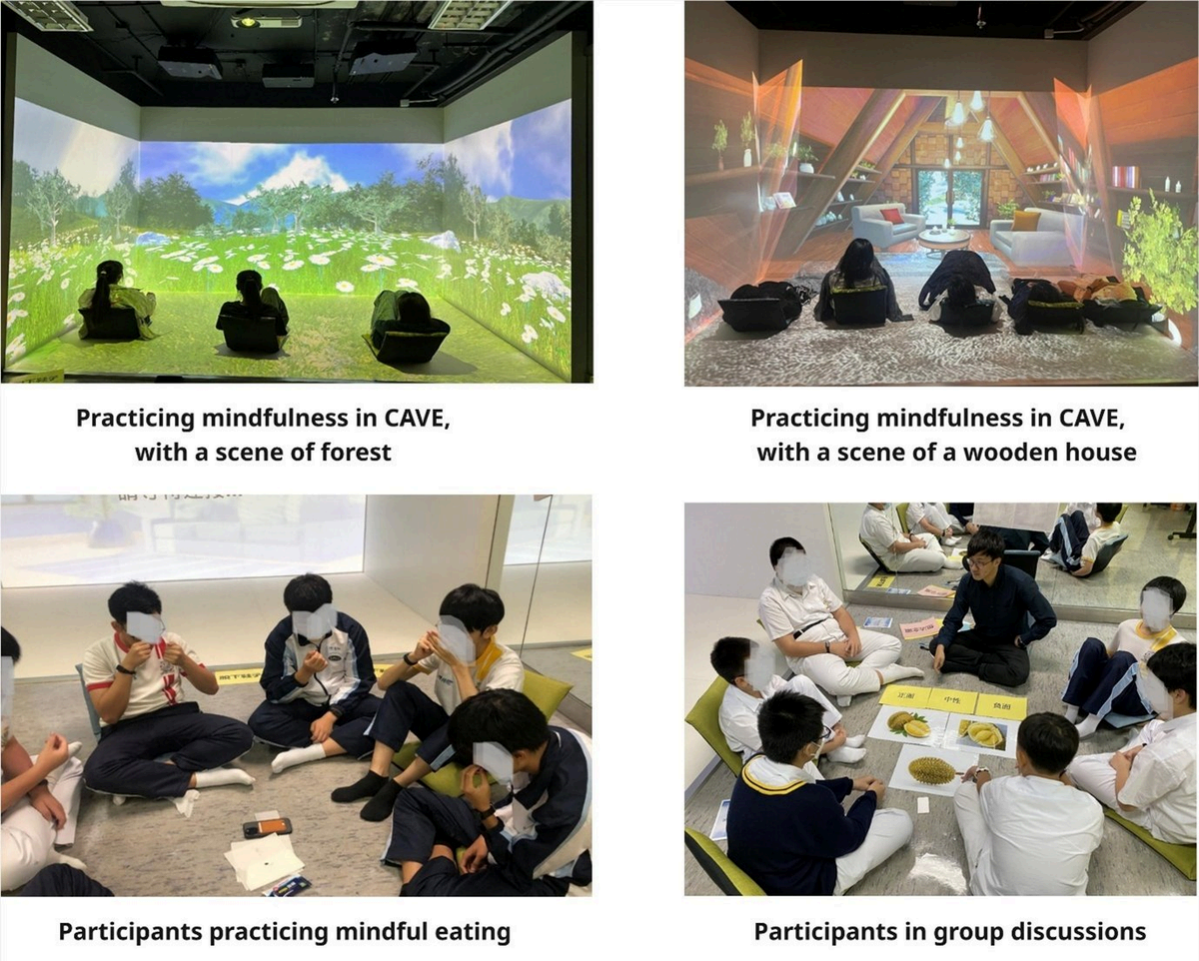
CAVE and key session activities.

The program comprised eight 1.5-hour group sessions delivered over 2 months and was conducted in a quiet room in a local youth service center in Hong Kong. It used a CAVE-based virtual reality system (240 cm depth×349 cm width×240 cm height) to create an immersive environment featuring natural scenes (eg, forests) and ambient sounds (eg, birdsong) to support mindfulness practice. Figure 1 illustrates the CAVE and key session activities.

Two social workers facilitated the sessions under the supervision of a clinical psychologist, with groups of approximately 6 participants. Prior to delivery, the social workers received intensive training provided by the supervising clinical psychologist, covering the theoretical underpinnings of the program (mindfulness-based cognitive therapy principles and mindful parenting framework), session structure, facilitation skills, and strategies for managing participants’ emotional responses. Throughout the intervention period, ongoing structured supervision and ad hoc supervision were also provided. Combined, these training and supervision arrangements totaled 60 hours and were designed to ensure the intervention was delivered as intended.

The scheduling of sessions was flexible and collaboratively arranged with participants and the interventionists, informed by our prior experience working with adolescents that flexible scheduling helps enhance attendance.

Each session had distinct learning objectives and key activities, as summarized in Table 1.

**Table 1.** Overview of the VR-MBI^a^ program.

Session	Objectives	Key activities
1	Introduction to mindfulness Distinguishing bodily sensations, feelings, and thoughts	Breathing space Experiential activity^b^ Mood diary introduction
2	Awareness of bodily sensations, feelings, and thoughts	Breathing space Experiential activity^b^ Perception exercise^c^ Mood diary sharing
3	Differentiating awareness from self-criticism Cultivating curiosity and acceptance	Breathing space Video-based reflection Guided discussion Mood diary sharing
4	Deepening embodied awareness Strengthening acceptance	Body scan Video-based reflection^d^ Exploration of thoughts and feelings Mood diary sharing
5	Acceptance and personal choice Introduction to self-compassion	Body scan Video-based reflection^d^ Discussion of habitual reactions Mood diary sharing
6	Awareness of habitual thought patterns Self-compassionate responding	Breathing space Guided imagery Video-based reflection^d^ Mood diary sharing
7	Intentional choice between awareness and self-criticism	Perception exercise^c^ Mindful eating Reflection on thoughts and feelings Mood diary sharing
8	Consolidation and motivation for ongoing practice	Body scan Group reflection Reflective letter writing

a VR-MBI: virtual reality mindfulness–based intervention.

b Examples of experiential activities included Pass a Cup (mindful observation of sensations) and See the World Blindfolded (paired sensory exploration).

c Examples of perception exercises included ambiguous images (eg, rabbit-duck illusion) to illustrate the flexible nature of attention and interpretation.

d Video-based reflections used short scenarios depicting adolescent stressors (eg, bullying) to facilitate discussion of thoughts, emotions, and habitual reactions.

#### Recruitment

We recruited participants through convenience sampling from a youth service organization and our secondary school networks in Hong Kong using promotional leaflets and social workers’ and teachers’ referrals. We contacted individuals who expressed interest in the study and invited them to a 10-minute in-person interview to assess eligibility. Those who met the inclusion criteria were provided with detailed information about the study and completed written informed consent, as outlined in the Ethical Considerations section above.

### Quantitative Data Collection

#### Feasibility and Acceptability

We assessed feasibility and acceptability using participant attendance, retention, homework practice frequency, dropouts, and adverse events (eg, emotional discomfort and distress). Homework data came from participants’ homework diaries, and all other data were systematically documented by the research team in research logs.

#### Effectiveness on Anxiety, Stress, and Depression

We assessed preliminary effectiveness on anxiety, depression, and stress using both subjective and objective measures.

#### Subjective Measures

We used a questionnaire comprising the Chinese versions of the Depression Anxiety Stress Scale–21 (DASS-21) and the Mindful Attention Awareness Scale (MAAS). The adult versions were selected because they are more widely used in the MBI literature, thereby facilitating cross-study comparability, and have demonstrated sound psychometric properties in Chinese adolescent samples [29-31].

The DASS-21 [32,33] includes 21 items rated on a 4-point scale to assess depression, anxiety, and stress over the past week. It produces 3 subscale scores on anxiety, depression, and stress (0‐21) and a total distress score (0‐63), with higher scores indicating greater symptom severity. In this study, the DASS-21 demonstrated excellent internal consistency at baseline (α=.94) and good internal consistency at follow-up (α=.90). Subscale reliabilities at baseline and follow-up were as follows: depression (α=.88 and .79), anxiety (α=.82 and .76), and stress (α=.84 and .72).

The MAAS assesses trait mindfulness using 15 items rated on a 6-point scale [34]. Scores represent the mean across items (1-6), with higher scores reflecting greater trait mindfulness. In the study, the MAAS showed high internal consistency at baseline (α=.91) and follow-up (α=.88).

All self-report measures, including the DASS-21 and MAAS, were compiled in a paper questionnaire and administered at baseline (T0, immediately before the first session) and postintervention (T1, within one week after the final session). Questionnaires were administered on-site at the intervention location by a trained research assistant who was independent of the intervention delivery.

#### Objective Measure

Heart rate variability (HRV), an objective indicator of autonomic stress regulation, was collected using the Upmood watch equipped with a 250 Hz photoplethysmogram sensor. HRV data were collected during the after-school period (4-6 PM) at both time points: at baseline (T0), prior to the completion of self-report questionnaires, and at postintervention (T1), following the final intervention session. A single recording window per time point was used to minimize participant burden, consistent with evidence that a single short-term recording yields valid estimates of standard deviation of normal-to-normal intervals (SDNN) and root-mean-square of successive differences (RMSSD) [35]. To ensure data quality and standardized device operation, research assistants completed a 5-hour structured training program prior to data collection, comprising a 2-hour session delivered by the Upmood technical team and an additional 3 hours of supervised practice.

#### Demographic Information

We also administered a preintervention questionnaire (Multimedia Appendix 1) capturing age, gender, family structure, number of coresidents, parental marital status, parental education levels, housing type, monthly family income (Hong Kong Dollars; HKD), and prior mindfulness experience (yes or no).

The questionnaire was administered on-site at the intervention venue prior to the first session by a trained research assistant who was independent of intervention delivery. Participants completed it individually, with the research assistant available to clarify items as needed.

### Qualitative Data Collection

We also conducted 1-hour postintervention semistructured group interviews with participants to gather feedback on the intervention. To promote open discussion and group cohesion, each interview group consisted of participants from the same mindfulness group (approximately 6 participants per group). A social worker and a research assistant, both independent of the intervention delivery, facilitated the interviews using an interview guide (Multimedia Appendix 1) that covered overall experiences, perceived changes, likes and dislikes, and challenges. All sessions were audio-recorded for transcription and analysis.

### Data Analyses

#### Quantitative Analysis

We conducted all statistical analyses in Stata 18 (StataCorp).

We summarized demographic characteristics and feasibility and acceptability outcomes using descriptive statistics (counts, percentages, means, and SDs). Attendance was calculated as the proportion of participants attending each session, with feasibility defined a priori as ≥70% of participants attending ≥80% (8/10) of sessions. Retention was reported as the proportion completing both baseline and postintervention assessments. Homework practice was summarized as the mean number of practices completed. Dropouts and adverse events were reported as counts and percentages.

To examine the effects of the intervention on distress, anxiety, depression, stress, and trait mindfulness, we used paired-sample *t* tests for normally distributed variables and Wilcoxon signed-rank tests for nonnormally distributed variables (2-tailed, *P*<.05). All assumptions relevant to the statistical tests were checked.

For HRV analysis, we used the HRV analysis with the R package and followed the recommended procedures described [36]. Pulse-to-pulse intervals were extracted from a 10-minute recording window. We removed artifacts using established threshold criteria (<300 ms or >2000 ms) [37] and resampled the time series at 4 Hz using a cubic spline [38]. We then calculated standard time-domain HRV metrics, including the SDNN and the RMSSD. We focused exclusively on time-domain measures, as time-domain indices are more robust to noise and more reliable for short recordings obtained from wearable devices [39,40].

SDNN reflects overall HRV, with higher values indicating greater overall autonomic flexibility and better stress regulation, whereas RMSSD reflects short-term HRV and parasympathetic activity, with higher values indicating more adaptive stress regulation.

Pre-post changes in HRV indices (RMSSD and SDNN) were analyzed using paired *t* tests or Wilcoxon tests, as appropriate (2-tailed, *P*<.05).

A sensitivity analysis was also conducted to examine whether prior mindfulness experience influenced intervention outcomes, including self-reported outcomes (distress, anxiety, depression, stress, and trait mindfulness) and HRV indices. Participants were divided into 2 groups: those with prior mindfulness experience and those without, and a 2×2 mixed ANOVA was performed to examine time × mindfulness experience interaction effects across all outcome measures (2-tailed, *P*<.05). Feasibility outcomes were additionally compared descriptively between the 2 groups, including the proportion of participants meeting the ≥80% attendance threshold and mean homework practice frequency.

#### Qualitative Analysis

A research assistant transcribed all group interviews verbatim. A research team member (CTKY) who has training in qualitative methods analyzed all transcripts using NVivo software (Lumivero) and Braun and Clarke’s reflexive thematic analysis [41,42] with an inductive approach. CTKY followed the 6 phases of reflexive thematic analysis: familiarization with the data, generation of initial codes, searching for themes, iterative reviewing and refinement of themes through discussion with other project team members (PPKK and APLT), defining and naming themes, and producing the report.

To enhance reflexivity, CTKY maintained analytic memos throughout the analysis and discussed emerging interpretations with the project team. These discussions helped examine assumptions and strengthen the credibility and rigor of the analysis.

#### Data Integration

We integrated quantitative and qualitative data at the interpretation stage, following a concurrent approach [43], in which we simultaneously explored the statistical patterns and in-depth meanings, with equal priority given to both data strands. We discussed findings from both sources using data triangulation, exploring convergence and divergence to compare and synthesize results, with integrated interpretations and a joint display table presented in the Results and Discussion section [44].

## Results

### Overview

**Figure 2.**
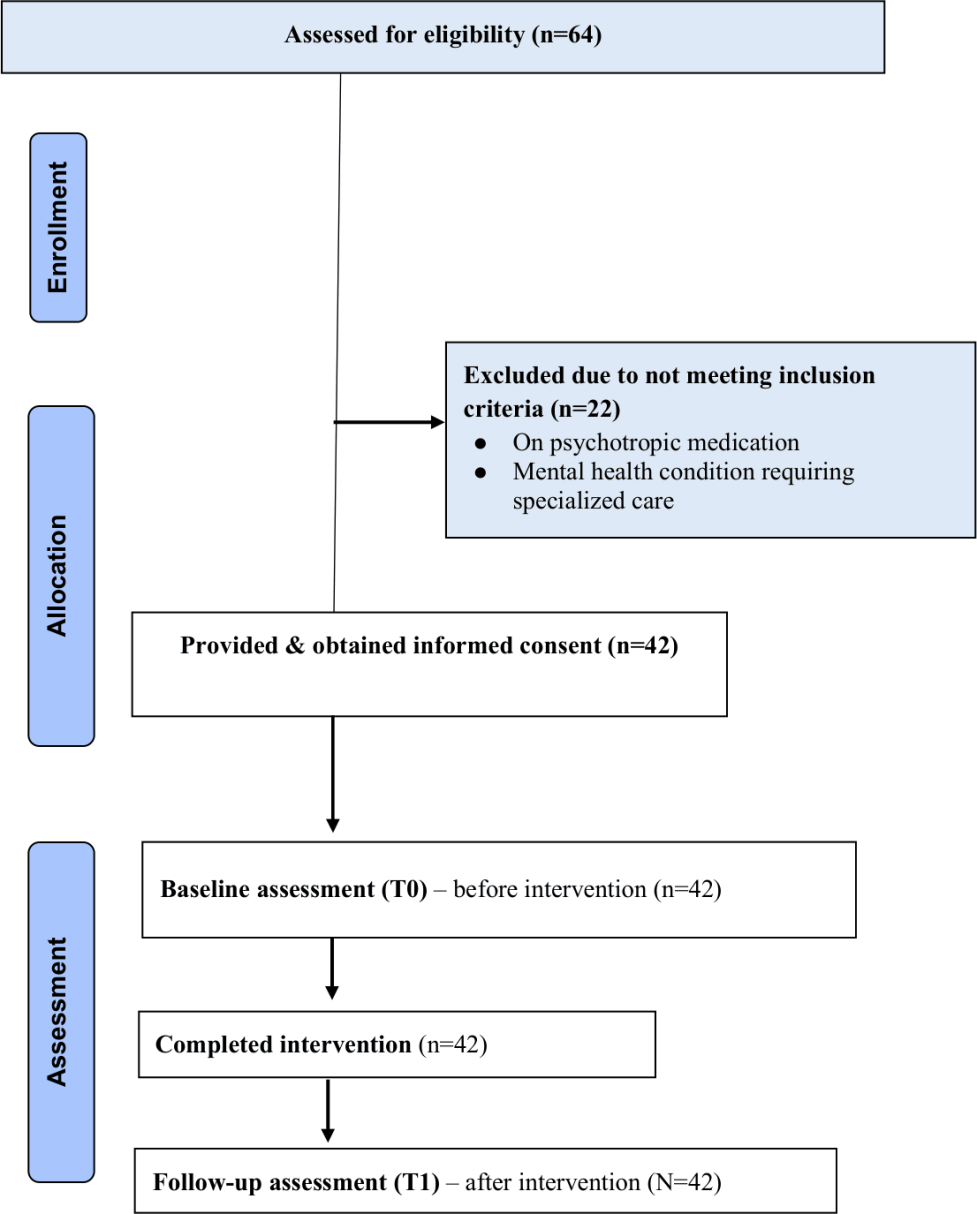
CONSORT (Consolidated Standards of Reporting Trials) diagram. HRV: heart rate variability.

We screened 64 potential participants, of whom 22 were excluded based on the predefined eligibility criteria. This resulted in 42 participants who were enrolled and provided informed consent. All enrolled participants completed the baseline assessment (T0), received the 8-week intervention, and completed the follow-up assessment (T1). Although all 42 participants wore the wearable device for HRV measurement at both time points, 9 had incomplete or unrecoverable recordings due to the wearable device failing to establish a stable Bluetooth connection (5 participants at T0 and 4 at T1); the paired HRV analysis was therefore conducted on 33 out of 42 (78.6%) participants only. Unless otherwise specified, all 42 participants were included in the analyses. Participant flow is illustrated in the CONSORT (Consolidated Standards of Reporting Trials) diagram (Figure 2).

In Figure 2, although all 42 participants wore the wearable device at T1, HRV data were available for 33 (78.6%) participants due to technical failure of the wearable device.

### Demographic Characteristics

Table 2 summarizes the demographic characteristics of the participants. The mean age was 14.88 (SD 1.90) years, and the gender distribution was balanced (20/42, 47.6% female and 22/42, 52.4% male). Most participants were from dual-parent families (27/38, 71.1%), and approximately 46% (16/35) came from households with a monthly family income at or below HKD 30,000 (approximately US $3800), which is the median household income in Hong Kong [45]. Approximately 64% (27/42) of participants reported prior experience with mindfulness practices.

**Table 2.** Demographic characteristics of the study participants (N=42)^a^.

	Values
Age (years), mean (SD)	14.88 (1.90)
Sex, n (%)	
Female	20 (47.6)
Male	22 (52.4)
Family structure	
Dual-parent family	27 (71.1)
Single-parent family	11 (28.9)
Monthly family income in HKD^b^	
<9,999 HKD^c^	5 (14.3)
10,000‐19,999 HKD^d^	5 (14.3)
20,000‐29,999 HKD^e^	6 (17.1)
30,000‐39,999 HKD^f^	8 (22.9)
40,000‐49,999 HKD^g^	3 (8.6)
≥50,000 HKD^h^	8 (22.9)
Previous experience in mindfulness practices, n (%)	
Yes	27 (64.3)
No	15 (35.7)

a Four participants (10%) did not respond to the family structure question, and 7 participants (17%) did not respond to the monthly family income question. Percentages reported are valid percentages, excluding missing data.

b Hong Kong Dollars.

c < US $1300.

d US $ 1300 ‐$ 2600.

e US $2600‐$3800.

f US $3800‐$5100.

g US $5100‐$6400.

h ≥ US $6400.

### Quantitative Findings

#### Feasibility and Acceptability

The study achieved a 100% retention rate, with all 42 participants completing both the intervention phase, pre- and postintervention self-report questionnaire assessments, and wearable device measurements at both time points. Although all 42 participants completed the wearable device measurements at T0 and T1, valid HRV recordings were obtained from 33 of 42 (78.6%) participants, as 9 participants had unrecoverable recordings due to technical failure (ie, Bluetooth disconnection) of the wearable devices.

Attendance was high, with 31 of 42 (74%) participants attending at least 80% (8/10) of the sessions. Participants also demonstrated engagement outside the sessions, with a mean homework practice count of 11 (SD 8) times within the 8-week period. No dropouts or adverse events were reported during the study period.

#### Effectiveness

Table 3 presents pre- to postintervention changes in DASS-21 subscale scores (depression, anxiety, and stress), total distress, self-reported trait mindfulness (MAAS), and HRV indices.

**Table 3.** Pre-post comparison results for psychological outcomes (DASS-21^a^ and MAAS^b^; N=42) and heart rate variability indices (n=33).

	Baseline (T0), mean (SD)	Postintervention (T1), mean (SD)	Mean difference (95% CI)	Statistics	*P* value	Effect size
Psychological outcomes						
Distress (DASS-21 total)	18.67 (13.52)	19.55 (11.27)	0.88 (−5.15 to 3.39)	0.732^c^	.46	.113^d^
Depression (DASS-21 subscore)	6.19 (5.51)	6.33 (4.37)	0.14 (−1.57 to 1.28)	0.586^c^	.56	.090^d^
Anxiety (DASS-21 subscore)	4.88 (4.15)	5.69 (4.08)	0.81 (−2.21 to 0.59)	1.803^c^	.07	.278^d^
Stress (DASS-21 subscore)	7.60 (4.90)	7.52 (4.20)	−0.07 (−1.72 to 1.86)	−0.081 (41)^e^	.94	.012^f^
Trait Mindfulness (MAAS)	64.64 (17.03)	62.62 (14.80)	−2.02 (−0.65 to 4.70)	−1.358^c^	.17^d^	.210
HRV^g^ Indices						
SDNN^h^ (ms)	131.60 (40.40)	149.19 (41.30)	17.60 (−33.88 to −1.32)	2.202 (32)^e^	.03^i^	.383^f^
RMSSD^j^ (ms)	146.16 (42.13)	166.36 (46.87)	20.20 (−38.76 to −1.65)	2.218 (32)^e^	.03^i^	.387^f^

a DASS-21: Depression Anxiety Stress Scale-21.

b MAAS: Mindful Attention Awareness Scale.

c *z* statistics from Wilcoxon signed-rank test.

d Effect size calculated using r (rank-biserial correlation), appropriate for non-parametric Wilcoxon signed-rank tests.

e *t* statistics from paired *t* test (2-tailed).

f Effect size calculated using Cohen *d*, appropriate for paired *t* test.

g HRV data were missing for 9 (21.4%) participants due to technical failure of the wearable device; the paired HRV analysis was therefore conducted on 33 (78.6%) participants.

h SDNN: Standard deviation of NN intervals. Higher SDNN reflects greater overall heart rate variability, indicating better stress regulation and greater physiological resilience.

i *P*<.05 (statistically significant).

j RMSSD: root-mean-square of successive differences. Higher RMSSD reflects stronger parasympathetic (vagal) activity, associated with the body’s ability to relax and recover from stress.

#### Self-Reported Psychological Outcomes

Results indicated no significant changes in self-reported psychological outcomes following the intervention. Total emotional distress remained stable from pre- to posttest (mean difference 0.88, 95% CI −5.15 to 3.39; *P*=.46). Similarly, no significant changes were observed in depression (mean difference 0.14, 95% CI −1.57 to 1.28; *P*=.56), anxiety (mean difference 0.81, 95% CI −2.21 to 0.59; *P*=.07), or stress (mean difference −0.07, 95% CI −1.72 to 1.86; *P*=.94). Self-reported trait mindfulness also did not change significantly (mean difference −2.02, 95% CI −0.65 to 4.70; *P*=.17). Full descriptive statistics and test statistics are presented in Table 3.

#### Changes in HRV Indices

In contrast, analyses of HRV indices revealed significant physiological changes following the intervention. Results showed a significant increase in SDNN from baseline to postintervention (mean difference 17.60 ms, 95% CI −33.88 to −1.32; *t*_32_=2.202; *P*=.04; Cohen *d*=.383). The RMSSD also increased significantly, from baseline to postintervention (mean difference 20.20 ms, 95% CI −38.76 to −1.65; *t*_32_=2.218; *P*=.03; Cohen *d*=.387). These findings may suggest enhanced parasympathetic activity and improved physiological stress regulation following the intervention.

#### Sensitivity Analyses

To examine whether prior mindfulness experience influenced intervention outcomes, a sensitivity analysis was conducted by dividing participants into those with (27/42, 64.3%) and without (15/42, 35.7%) prior mindfulness experience.

A series of 2×2 mixed ANOVAs was conducted with time (pre- vs postintervention, within-subjects) and mindfulness experience (between-subjects) as factors across all outcomes (DASS-21 total and subscale scores, MAAS, and HRV indices). No significant time×mindfulness experience interaction effects were observed across any outcome, suggesting that prior mindfulness experience did not meaningfully affect pre-to-post changes, lending confidence that the observed outcomes were not attributable to participants’ prior familiarity with mindfulness practice. Detailed results are presented in Multimedia Appendix 2.

For feasibility outcomes, attendance rates were comparable between the two groups, with 87% (13/15) of participants without prior experience and 67% (18/27) of participants with prior experience meeting the ≥80% attendance threshold. Homework practice frequency was similarly comparable across groups (without prior experience: mean 13.27, SD 7.38; with prior experience: mean 10.19, SD 7.71).

### Qualitative Findings

All participants (N=42) attended the focus group interviews. Analysis of the focus group interviews identified 7 themes, which were organized into three overarching categories: (1) perceived mental health and well-being benefits (themes 1‐3), (2) facilitators of the intervention (theme 4), and (3) implementation challenges (themes 5‐7).

#### Theme 1: Mindfulness Supports Regulation of Negative Emotions

Participants described mindfulness as a practical strategy for regulating negative emotions, particularly stress and anger, by creating a pause that allowed them to respond more calmly in challenging situations.

Several participants emphasized how mindfulness helped them manage interpersonal conflicts by reducing impulsive reactions. One participant reflected on family arguments, noting a shift from reacting defensively to listening and letting go:

*In the past, I might say a sentence or two … Now I choose to just listen and let it go. I still feel angry inside… but I will practice mindfulness… Now I feel it’s better*.

[F7-2; Female]

The same participant described applying mindfulness when feeling irritated while teaching her younger sister, using the practice to calm herself before responding:

*If I really feel like scolding her, I’ll practice once first, then ask someone else to teach her, and only when I’ve calmed down will I go back to teach her*.

[F7-2; Female]

This capacity to pause and reflect before acting was also evident in school settings. One participant involved in a school committee noted that mindfulness helped prevent impulsive decisions during conflict:

*I think if I hadn’t joined the mindfulness sessions, I probably would have just not done it (continued with the school committee position) … But now… I would stop and think about it first*.

[F3-2; Male]

Others described reduced anger and aggressive urges in everyday situations, such as gaming frustration:

*After doing it, I felt more relaxed, less angry… less like wanting to hit those teammates.*

[F1-5; Male]

and

*After doing it, my mood improves… my emotions calm down… (before) I would hit a pillow.*

[F7-4; Female]

#### Theme 2: Mindfulness Fosters Emotional Awareness and Self-Understanding

Participants described mindfulness as enhancing their awareness of emotions, thoughts, and bodily sensations, which in turn supported greater self-understanding and emotional clarity. One participant contrasted mindfulness with earlier stress-management approaches taught in school:

*After practicing mindfulness, I became more aware of my thoughts and emotions. Back in primary school, they only told us to relax and not give ourselves too much pressure, but they never taught us how to actually notice our emotions and thoughts.*

[F7-1; Female]

Others reflected on how this increased awareness reduced feelings of confusion and pessimism about their daily lives. One participant described gaining a clearer sense of self and a more positive outlook over the course of the program:

*Eight weeks ago, I might have felt somewhat lost about school life or about myself. But over these eight weeks, I feel I’ve gained more understanding—understanding myself better, and maybe not feeling as pessimistic about school life as before*.

[F1-4; Male]

Mindfulness practices also enhanced awareness of bodily sensations, helping participants recognize physical discomfort and needs more clearly. One participant highlighted the impact of body scan exercises:

*During the body scan, you pay more attention to whether each part of the body feels uncomfortable, which makes it clearer where you might have needs.*

[F5-2; Female]

#### Theme 3: Mindfulness Enhances Focus and Sleep Quality

Participants frequently reported improved focus following mindfulness practice, describing feeling more attentive and less distracted during and after the sessions. Some expressed this briefly:

*I can concentrate better.*

[F5-1; Female]

and

*My attention has also improved a bit.*

[F5-5; Male]

Others highlighted that mindfulness created a mental space that allowed them to disengage from academic pressures:

*Each time I come here, I can have an hour and a half to stay focused on this and get away from my usual academic pressure… once you’re engaged in it, you won’t be thinking about those things*.

[F6-2; Female]

In addition to improved focus, many participants reported better sleep quality after learning mindfulness techniques. Several described intentionally practicing mindfulness before bedtime to help them fall asleep, including one participant who shared:

*I used to have insomnia about four or five times a week… I’ll practice [mindfulness], and it helps me fall asleep.*

[F5-2; Female]

And another who noted:

*My sleep quality has improved a bit… so I use it to help me sleep.*

[F5-5; Male]

#### Theme 4: The Immersive CAVE Environment and Group-Based Format Facilitated Engagement and Motivation

Participants identified the immersive CAVE environment as a key facilitator of engagement in mindfulness practice. The CAVE was frequently described as a distraction-free space that supported sustained attention. The simulated projected nature scenes, together with the sound, made the experience more immersive.

*When I close my eyes and calm my mind, I feel as if I am truly in that environment, because the sounds are very realistic…I became more focused.*

[F1-1; Male]

*I prefer to be in the CAVE — it’s very immersive. There are some simulated scenes, some lighting, and it projects images of grassland and nature, which gives a very relaxing feeling*.

[F3-2; Male]

Several participants contrasted practicing mindfulness in the CAVE with doing so at home or at school, noting that the quiet environment made it easier to concentrate:

*At home it’s noisy, but in the CAVE it’s very quiet, so when I practice mindfulness there, I can focus more.*

[F1-5; Male]

*I think doing it here is actually better than doing it at home, because here everyone is doing mindfulness together, and in that quiet environment, just listening to the sounds gradually calms you down and helps you focus. At home there might be books or other things around that distract me.*

[F2-1; Female]

Others similarly noted:

*At school, with so many people around, it’s hard to get into a mindfulness state*.

[F4-3; Male]

Another participant compared phone-based mindfulness practice with the CAVE, noting that:

*The phone screen is too small (when using mindfulness app on phone)… this (CAVE) feels more real… It’s like actually being inside it*.

[F2-3; Female]

In addition to the physical environment of the CAVE, participants emphasized the motivational benefits of practicing mindfulness in a group in the CAVE. Many reported that the presence of others helped them stay engaged:

*Having someone there allows me to concentrate better and really immerse myself*.

[F1-1; Male]

and

*Here there are more people accompanying me… having more people around feels better.*

[F3-1; Male]

The group setting also fostered social connection and stress relief, with participants describing shared practice as an opportunity to:

*Relieve the stress*.

[F1-4; Male]

and

*Build trust through sharing experiences.*

[F5-3; Male]

#### Theme 5: Technological Limitations Disrupted Mindfulness Practice

Participants reported that certain technological aspects of the CAVE occasionally disrupted their mindfulness experience. Several described the on-screen visuals as unnatural or lacking realism, which reduced immersion and distracted them during practice. One participant explained:

*The first time I watched it, I felt it was a bit fake… when you look up close, it gets pixelated.*

[F3-3; Male]

Another noted:

*If I look at the screen and it doesn’t feel very real, I’ll just judge it as something fake, and that distracts me.*

[F5-3; Male]

Brightness and visual intensity were also reported as barriers. Some participants found the screens uncomfortably glaring, which interfered with relaxation:

*What feels uncomfortable in the CAVE is that it’s too bright.*

[F2-3; Female]

and

*The screen is too glaring, and the colors are too bright.*

[F5-1; Female]

#### Theme 6: Difficulty Applying Mindfulness During Intense or Persistent Emotions

Some participants described challenges in applying mindfulness when emotions, particularly anger, were intense or long-lasting. In such moments, emotional reactions felt automatic and difficult to regulate. One participant acknowledged reverting to previous coping patterns when emotions became overwhelming:

*When I really ‘can’t handle it,’ I’ll go back to the old way*.

[F2-1; Female]

Others noted that while mindfulness helped reduce distress in the moment, its effects were sometimes temporary. One participant reflected:

*Right after that incident happened, I did mindfulness and felt calmer… but once I thought back to the incident, the anger would come up again*.

[F1-1; Male]

#### Theme 7: Daily Life Constraints Limited Integration of Mindfulness Practices

Participants also described practical barriers to integrating mindfulness into daily routines, particularly for practices such as mindful eating. Time constraints were frequently cited, with participants feeling that mindfulness practices were difficult to sustain amid busy schedules. One participant explained simply:

*No time.*

[F1-1; Male]

while another elaborated:

*Looking at one piece of food took too long, and usually I have to eat quickly because of the limited lunch hour at school*.

[F1-2; Female]

Family expectations further constrained practice, especially when mindfulness behaviors conflicted with household routines. One participant noted:

*My parents would scold me as I am eating too slow (during dinner)”* when attempting mindful eating.

[F1-1; Male]

### Integration of Quantitative and Qualitative Findings

Table 4 presents a joint display integrating the quantitative and qualitative findings. Quantitative outcomes are presented alongside corresponding qualitative themes and illustrative quotes, with convergent and divergent patterns identified; integrated interpretations are discussed in the Discussion section.

**Table 4.** Joint display integrating quantitative and qualitative findings.

Quantitative findings	Qualitative themes	Convergent^a^ or divergent^b^	Inference
Feasibility and acceptability (primary outcome)			
High attendance and retention with homework practice	The immersive environment and group format enhanced engagement, though technological limitations posed challenges	Convergent	Strong attendance and retention corroborate the CAVE and group format as key facilitators, though technological limitations indicate room for refinement. Daily life constraints contextualize variability in homework practice.
Preliminary effects on psychological well-being (secondary outcomes)			
No improvement in stress, anxiety, or depression on self-report measures	MBI^c^ regulates negative emotions MBI is less effective when emotions are intense	Divergent	Every day, emotional regulation benefits were real but insufficient after 8 weeks to shift scale scores or sustain regulation under intense emotions, suggesting an inadequate intervention dose.
Improvement in HRV indices^d^	MBI fostered calmer emotional responding and greater awareness of thoughts and feelings	Convergent	Enhanced emotional awareness provides a plausible mechanism for the observed physiological stress regulation gains.
No improvement in trait mindfulness	Mindfulness fostered greater awareness of thoughts and feelings and the ability to concentrate	Divergent	Qualitative gains in awareness and focus suggest real state-level benefits not yet detectable by the MAAS^e^ as a trait-level measure over 8 weeks.

a Convergent: quantitative and qualitative findings point in the same direction.

b Divergent: findings differ across data strands.

c MBI: Mindfulness-based interventions.

d HRV: Heart rate variability indices, including standard deviation of normal-to-normal intervals and root-mean-square of successive differences.

e MAAS: Mindful Attention Awareness Scale.

## Discussion

### Principal Findings

This study examined the feasibility, acceptability, and preliminary efficacy of a CAVE-based VR-MBI for adolescents with mild-to-moderate anxiety in Hong Kong. Our findings suggested that the intervention demonstrated strong feasibility and acceptability, as evidenced by high attendance, full retention, active engagement in home practice, and the absence of adverse events. Qualitative findings further supported these outcomes, with participants reporting positive experiences and perceived benefits in stress reduction, sleep quality, concentration, and self-awareness. Key facilitators of engagement included the distraction-free CAVE environment and the motivational benefits of shared group practice. Barriers included technological limitations such as pixelation and screen brightness, difficulties applying mindfulness during intense emotions, and practical daily life constraints, particularly time pressures, that limited the integration of mindfulness into everyday routines.

Regarding preliminary efficacy, no significant pre-post changes were found in self-reported distress, anxiety, depression, stress, or trait mindfulness. However, preliminary improvements in both HRV indices (SDNN and RMSSD) may suggest enhanced physiological stress regulation postintervention.

### Comparison With Existing Studies and Implication

#### Feasibility and Acceptability

To the best of our knowledge, this study represents the first local evaluation of an immersive virtual reality MBI for adolescents in Hong Kong. Our findings indicate high feasibility and acceptability of VR-MBI for adolescents, which aligns with prior research conducted in other countries. For example, the “Relaxing Environment for Stress in Teens” (RESeT) program in the United States reported strong engagement and good usability, along with high acceptability, feasibility, and appropriateness ratings among adolescents. Similarly, adolescents in the RESeT program also highlighted the immersive VR environment as a key factor supporting engagement and temporary disengagement from distressing thoughts and emotions [24].

Our VR-MBI appears to demonstrate better feasibility and acceptability than traditional mindfulness interventions previously evaluated in Hong Kong. A local Hong Kong study reported moderate acceptability, with only about half of participants rating the program positively and very low home practice rates [12]. Similarly, a school-based MBI local program observed high dropout rates (41%), low attendance (50%), behavioral issues during sessions, limited engagement, and minimal home practice [46]. The immersive CAVE environment may have been an important factor underlying this discrepancy. VR has been empirically demonstrated to enhance immersion and sustained attention during practice [17-19], and our qualitative findings corroborate this—participants consistently identified the CAVE environment as a key facilitator of engagement, describing it as a comfortable, distraction-free space that was qualitatively distinct from other mindfulness delivery modes such as home-based practice, smartphone apps, and classroom-based delivery. That said, we acknowledge that other contextual and design factors likely also contributed. The challenges reported in prior local studies, including large class sizes, compulsory participation, restrictive classroom environments, and monotonous activities [46], were largely addressed through deliberate program design choices in this study, including small group sizes, interactive and creative activities, and flexible scheduling arranged collaboratively with participants. The improved feasibility and acceptability observed in this study, therefore, likely reflects the combined contribution of the immersive VR environment and these broader design considerations.

Despite these strengths, our study identified several VR technologies–related challenges that warrant further refinement to enhance feasibility and acceptability. Although the CAVE was deliberately designed as a headset-free VR system to minimize physical discomfort such as dizziness, participants still reported limitations related to the visual design and display characteristics of the system. Specifically, some noted that the visuals appeared unnatural and that the screens were overly bright, which reduced immersion and interfered with relaxation during mindfulness practice. Similar issues have been reported in other VR-based mindfulness studies, where sensory discomfort and image quality concerns have emerged when VR environments are not optimally designed [47,48]. Our participants also reported that they faced challenges in using mindfulness when emotions were very intense, noting that the practice offered limited benefit in such moments. This aligns with prior research highlighting similar challenges [49,50], and it is suggested that integrating complementary strategies (eg, cognitive reappraisal, grounding) or offering brief “mini practices” may provide additional support. Our participants also described difficulty sustaining certain practices outside sessions, such as mindful eating, due to time constraints and limited family support. Similar barriers have been reported among adolescents more broadly, who often struggle to maintain regular practice without external support [12]. To address this, prior studies recommend embedding short practices into daily routines and involving family members in the intervention to help sustain motivation and continuity [28,51]. Addressing these challenges in future iterations is important for optimizing the feasibility and acceptability of VR-MBI for adolescents.

#### Effectiveness

Preliminary improvements in physiological stress regulation (SDNN and RMSSD) were observed post intervention. These positive changes were complemented by qualitative findings, in which participants described reduced stress, increased calmness, and improved emotion regulation. This finding is consistent with a review reporting improvement in SDNN and RMSSD following 8 weeks of mindfulness training [40], as well as with similar findings observed in MBIs’ randomized controlled trials targeting adolescents [52]. One proposed mechanism underlying these HRV improvements is that mindfulness practice often involves slow, attentive breathing, which promotes parasympathetic (vagal) activation, enhances physiological relaxation, and improves the body’s capacity to regulate stress [53].

However, contrary to expectations, the intervention did not result in improvement in self-reported anxiety, depression, stress, or trait mindfulness. One plausible explanation is a potential floor effect [54]. Our post hoc examination indicated that only 38% (16/42) of participants reported clinically elevated anxiety on the DASS-21 anxiety subscale at baseline (mean: 9.19, SD: 3.29), whereas the remaining 62% (26/42) scored below the clinical threshold (mean: 2.23, SD: 1.61), indicating limited room for measurable improvement at the group level. This interpretation is further supported by qualitative accounts in which participants described applying mindfulness as an active strategy to manage acute stress responses in emotionally challenging situations (theme 1), consistent with the notion that those with greater baseline distress may derive more measurable benefit, while those with lower baseline scores have less capacity for detectable change. We acknowledge, however, that this remains a post hoc interpretation and should be examined more rigorously in future studies with a larger sample size stratified by baseline anxiety severity.

The mixed findings, where the VR-MBI was effective in improving objective HRV indices but not subjective self-report measures, may be plausibly explained by differences in the dimensions of psychological functioning captured by these outcomes. HRV indices are sensitive to short-term changes in autonomic regulation and may therefore detect early physiological effects of mindfulness practice [39,55]. In contrast, the DASS-21 and MAAS primarily assess broader and more stable mood states and trait-like characteristics, which may require longer intervention exposure or sustained practice before significant changes become detectable. This interpretation aligns with prior evidence. A meta-analysis of MBIs in children and adolescents reported a trend toward reductions in self-reported anxiety symptoms, but effect sizes were small and nonsignificant [56]. Mechanistic accounts further propose that early gains from mindfulness are most evident in domains of emotion regulation and stress reactivity, while more stable improvements in depression and anxiety typically require longer duration or greater intensity of practice [57]. From this perspective, our 8-week VR-MBI may have facilitated short-term physiological and subjective improvements in well-being, while more enduring shifts in mood states would likely require extended or repeated engagement with practice. However, this interpretation requires further investigation in future studies.

Taken together, these findings suggest that our participants may have experienced meaningful early benefits, evident in physiological regulation and qualitative reports, that were not yet sufficiently robust to be reflected in self-reported mood measures. Future programs may consider extended duration or booster sessions. Our study also highlighted the importance of using a mixed methods design by integrating quantitative self-report measures, objective physiological biomarkers of stress regulation, and qualitative findings. Future studies should adopt similar approaches, as this can provide a nuanced understanding of efficacy, including capturing changes not fully captured by subjective measures or objective measures alone.

### Limitations

This study has several limitations. First, the pre-post, single-group design without a control group limits causal inference and precludes definitive conclusions about both the feasibility and effectiveness of the intervention. Without a comparator condition, it is unclear whether the observed feasibility outcomes, such as high attendance and retention, are specifically attributable to the VR-MBI. Similarly, observed improvements in HRV indices may be confounded by factors such as natural maturation, measurement variability, or nonspecific effects such as novelty of the activity and social interactions. All findings should be interpreted as preliminary, and no causal conclusions can be drawn. Additionally, the unique contribution of the CAVE cannot be empirically disentangled from other intervention components, such as the small-group format, skilled facilitation, and interactive activities, as no comparison condition was included. A future RCT incorporating both an active control condition (eg, a matched group program without VR) and a waitlist or treatment-as-usual control group would be necessary to establish both feasibility and efficacy more definitively and isolate the specific contribution of the CAVE technology.

Second, the sample size was relatively small (N=42), and a substantial proportion of participants (27/42, 64%) reported prior mindfulness experience. Participants with previous exposure to mindfulness who voluntarily enrolled may have been more positively predisposed toward mindfulness practice, and prior research suggests that mindfulness benefits may differ between experienced and novice practitioners [58]. Although a sensitivity analysis found no differences in feasibility or effectiveness outcomes between those with and without prior experience, the relatively small sample size within each subgroup limits the statistical power to detect such differences. Consequently, the generalizability of findings to adolescents with no prior mindfulness experience warrants caution and should be examined in future studies with larger samples.

Third, outcomes were assessed only immediately post intervention; thus, the durability of effects on anxiety, depression, and stress regulation remains unknown.

Fourth, while inclusion criteria required mild-to-moderate anxiety symptoms based on GAD-7 scores (>10) or professional referral, only 38% (16/42) of participants presented with clinically elevated anxiety on the DASS-21 at baseline. This discrepancy likely reflects two factors. First, our recruitment data show 54.8% of our participants (23/42) were recruited via professional referral with subthreshold GAD-7 scores (≤10), consistent with evidence that social workers and teachers can identify clinically meaningful distress and functional impairment not always captured by standardized measures [59,60], and may still warrant intervention despite subthreshold scores. Second, the GAD-7 and DASS-21 anxiety subscales assess partially overlapping but distinct constructs, the GAD-7 targeting generalized worry and the DASS-21 emphasizing physiological and panic-related symptoms, and demonstrate only moderate cross-instrument agreement [61]. Together, these factors may account for the observed discrepancy. Nevertheless, the sample may reasonably be considered to represent adolescents with anxiety symptoms, given the GAD-7’s greater sensitivity in detecting generalized anxiety disorder [61] and the capacity of professional referrals to capture clinically meaningful distress beyond what standardized measures alone may detect [59,60].

Fifth, the CAVE system requires installation of three projection walls, which may limit applicability in resource-constrained settings. Alternative formats such as VR head-mounted displays and mobile VR offer greater portability and accessibility, potentially overcoming this limitation. However, VR head-mounted displays are commonly associated with cybersickness symptoms, including headaches and nausea [24], which may compromise engagement particularly in adolescents, while mobile VR may offer insufficient immersion due to smaller screen sizes, limitations that the CAVE system specifically addresses. Future technological development should therefore aim to design compact, accessible VR devices that maintain adequate immersion while minimizing cybersickness risk.

Finally, HRV data were unavailable for 9 participants due to Bluetooth signal dropout of the wearable device. Although this was attributable to a technical failure, the possibility of systematic differences between those with and without valid recordings cannot be entirely ruled out, which may have introduced some degree of bias in the HRV findings.

### Conclusion

This study provides the first local evaluation of an immersive VR-MBI for adolescents in Hong Kong and offers preliminary evidence of its feasibility and acceptability. High engagement and positive qualitative feedback highlight the potential of CAVE-based VR to address common barriers associated with traditional mindfulness delivery.

Although self-reported psychological outcomes did not show significant change, we observed preliminary improvements in physiological indicators of stress regulation, together with participants’ subjective experiences, suggesting that VR-MBI may confer early benefits not fully captured by self-report measures. These findings underscore the promise of immersive VR as an innovative delivery modality for adolescent mindfulness interventions and support continued investigation using more rigorous study designs. But this should be interpreted with caution, given the study’s single-group design and absence of a control condition. Future studies should employ larger and more diverse samples, controlled or randomized designs with active and passive control groups, and extended follow-up periods to examine both short- and long-term outcomes. The inclusion of multimodal assessment approaches, such as objective biomarkers of stress regulation (eg, HRV), may help capture intervention-related changes that are not fully reflected in self-report measures.

From an implementation perspective, CAVE-based VR-MBI programs may represent a promising approach for youth mental health services in school and community settings. To optimize implementation, future programs should ensure high-quality, user-tested immersive environments, retain a group-based and interactive format, and integrate additional strategies to support adolescents’ regulation of intense emotions and application of mindfulness skills in daily life (eg, cognitive reappraisal and brief mini-practices). Encouraging home practice with family involvement, as well as extending program duration or offering booster sessions, may further support skill consolidation and sustained benefits.

## Supplementary material

10.2196/91819 Multimedia Appendix 1 Data collection tools (demographics questions and interview guideline).

10.2196/91819 Multimedia Appendix 2 Sensitivity analysis: outcomes by prior mindfulness experience.

10.2196/91819 Checklist 1 TREND checklist.

## Abbreviations

CONSORT: Consolidated Standards of Reporting Trials
DASS-21: Depression Anxiety Stress Scale–21
GAD-7: Generalized Anxiety Disorder–7
HRV: heart rate variability
MAAS: Mindful Attention Awareness Scale
MBI: mindfulness-based intervention
RESeT: Relaxing Environment for Stress in Teens
RMSSD: root-mean-square of successive differences
SDNN: standard deviation of normal-to-normal intervals
VR: virtual reality
