# Supplementary material for: Using a Virtual Reality CAVE–Based Mindfulness Intervention to Promote Mental Well-Being in Adolescents With Anxiety Symptoms: Pre-Post Mixed Methods Pilot Study
Source: JMIR Form Res. 2026 Jun 12;10:e91819. doi: 10.2196/91819 (PMC13263010; doi:10.2196/91819)
Supplement: Multimedia Appendix 1 [file formative-v10-e91819-s001.docx]

## **Multimedia Appendix- Data collection tools (Demographics questions, Interview guideline)**

## **Demographic information Questionnaire**

*Please tick (☑) the box that best describes you or fill in the blank where applicable.*

**1. Age**

Age: ___________________________

**2. Gender**

☐ Female

☐ Male

☐ Other (please specify): _______________

**3. Family Structure**

Which of the following best describes your current family arrangement?

☐ Dual-parent family (living with both parents)

☐ Single-parent family (living with one parent)

☐ Other (please specify): _______________

**4. Number of People Living in Your Household**

Not including yourself, how many people currently live with you?

Number of co-residents: ___________________________

**5. Parental Marital Status**

What is the current marital status of your parents?

☐ Married

☐ Not married / Never married

☐ Separated

☐ Divorced

☐ Widowed

☐ Other (please specify): _______________

**6. Father's Highest Education Level**

☐ Primary school or below

☐ Secondary school

☐ Tertiary education or above (e.g., university, college)

☐ Not applicable / Unknown

**7. Mother's Highest Education Level**

☐ Primary school or below

☐ Secondary school

☐ Tertiary education or above (e.g., university, college)

☐ Not applicable / Unknown

**8. Current Housing Type**

☐ Public rental housing

☐ Private rental housing

☐ Private property (owned)

☐ Other (please specify): _______________

**9. Monthly Family Income (HKD)**

What is your family's approximate monthly income?

☐ Less than HKD 9,999

☐ HKD 10,000 – 19,999

☐ HKD 20,000 – 29,999

☐ HKD 30,000 – 39,999

☐ HKD 40,000 – 49,999

☐ HKD 50,000 or above

☐ Prefer not to say

## **Interview Guide**

This semi-structured interview guide was used to explore participants’ experiences of the VR-based mindfulness intervention (VR-MBI). Each focus group interview lasted approximately one hour and included three to six participants, grouped according to their VR-MBI session allocation. The guide covered domains such as overall experience, perceived changes, helpful and challenging aspects of the intervention, and comparisons with other anxiety management strategies.

**Interview Questions**

1. Can you describe your overall experience with the VR-based MBI for anxiety? What were your thoughts and feelings before, during, and after using it?
2. Did you find the virtual reality environment engaging and immersive? How did it contribute to your ability to focus and stay present during the mindfulness exercises?
3. Did you notice any specific changes in your anxiety levels after the VR-based MBI? If so, can you describe those changes?
4. Were there any particular aspects of the VR mindfulness intervention that you found helpful or effective in managing your anxiety? Could you provide examples?
5. Were there any challenges or limitations you encountered in the VR-based MBI? How did those challenges affect your experience or the potential benefits?
6. How would you compare the VR-based MBI to other anxiety management techniques you’ve tried in the past? What sets it apart?
7. How likely would you be to recommend this VR-based MBI to your friends dealing with anxiety? Why or why not?
