# Supplementary material for: Using a Virtual Reality CAVE–Based Mindfulness Intervention to Promote Mental Well-Being in Adolescents With Anxiety Symptoms: Pre-Post Mixed Methods Pilot Study
Source: JMIR Form Res. 2026 Jun 12;10:e91819. doi: 10.2196/91819 (PMC13263010; doi:10.2196/91819)
Supplement: Multimedia Appendix 2 [file formative-v10-e91819-s002.docx]

## **Multimedia Appendix: Sensitivity Analysis: Outcomes by Prior Mindfulness Experience**

To examine whether prior mindfulness experience confounded study outcomes, a series of 2×2 mixed ANOVAs were conducted with Time (pre- vs post-intervention, within-subjects) and Mindfulness Experience (with vs without, between-subjects) as factors. Outcomes included DASS-21 total and subscale scores, trait mindfulness (MAAS), and HRV indices (SDNN and RMSSD). Of the 42 participants, 15 (35.7%) had no prior mindfulness experience and 27 (64.3%) had prior experience. Results are presented in the table below

No significant main effects of prior mindfulness experience, nor Time × Mindfulness Experience interaction effects, were observed across any outcome, suggesting that prior mindfulness experience did not meaningfully influence pre-to-post changes in the intervention.

| Table. 2×2 Mixed ANOVA Results: Time × Mindfulness Experience | | | | | | | | | | | | | |
| --- | --- | --- | --- | --- | --- | --- | --- | --- | --- | --- | --- | --- | --- |
| Outcome | Without Prior experience (n=15) | |  | With Prior experience (n=27) | |  | Group effect (Mindfulness Experience Yes/No) | | Time effect  (Pre vs Post) | | | Interaction effect | |
|  | Pre  *Means* | Post  Means |  | Pre  *Means* | Post  Means |  | *F* (1,40) | *p* | | *F*(1,40) | *p* | *F*(1,40) | *p* |
| DASS-Depression | 5.13 | 5.53 |  | 6.78 | 6.78 |  | 1.03 | .315 | 0.07 | | .789 | 0.07 | .789 |
| DASS-Anxiety | 4.13 | 4.93 |  | 5.30 | 6.11 |  | 1.11 | .298 | 1.22 | | .277 | 0.00 | .992 |
| DASS-Stress | 7.07 | 6.33 |  | 7.89 | 8.19 |  | 1.38 | .247 | 0.05 | | .816 | 0.31 | .584 |
| DASS-Total | 16.33 | 16.80 |  | 19.96 | 21.07 |  | 1.41 | .242 | 0.12 | | .726 | 0.02 | .886 |
| MAAS | 65.47 | 65.00 |  | 64.19 | 61.30 |  | 0.25 | .621 | 1.46 | | .233 | 0.76 | .388 |
| SDNN | 130.44 | 142.98 |  | 126.92 | 151.01 |  | 0.34 | .562 | 3.01 | | .093 | 0.48 | .493 |
| RMSSD | 152.59 | 167.76 |  | 138.69 | 167.52 |  | 0.01 | .914 | 2.93 | | .097 | 0.64 | .431 |
